# Mesenchymal stem cells use extracellular vesicles to outsource mitophagy and shuttle microRNAs

**DOI:** 10.1038/ncomms9472

**Published:** 2015-10-07

**Authors:** Donald G. Phinney, Michelangelo Di Giuseppe, Joel Njah, Ernest Sala, Sruti Shiva, Claudette M. St Croix, Donna B. Stolz, Simon C. Watkins, Y. Peter Di, George D. Leikauf, Jay Kolls, David W. H. Riches, Giuseppe Deiuliis, Naftali Kaminski, Siddaraju V. Boregowda, David H. McKenna, Luis A. Ortiz

**Affiliations:** 1 Department of Molecular Therapeutics, The Scripps Research Institute, Jupiter, Florida 33458, USA; 2 Department of Environmental and Occupational Health, University of Pittsburgh, Pittsburgh, Pennsylvania 15219, USA; 3 Hospital Son Espases, Palma Mallorca 07010, Spain; 4 Department of Pharmacology, University of Pittsburgh, Pittsburgh, Pennsylvania 15219, USA; 5 Department of Cell Biology, University of Pittsburgh, Pittsburgh, Pennsylvania 15219, USA; 6 Mellon Foundation Institute for Pediatric Research, University of Pittsburgh, Pittsburgh, Pennsylvania 15219, USA; 7 Department of Pediatrics, National Jewish Health, Denver, Colorado 80206, USA; 8 Department of Medicine, Yale University, New Haven, Connecticut 06510, USA; 9 Department of Laboratory Medicine and Pathology, University of Minnesota, Saint Paul, Minnesota 55108, USA

## Abstract

Mesenchymal stem cells (MSCs) and macrophages are fundamental components of the stem cell niche and function coordinately to regulate haematopoietic stem cell self-renewal and mobilization. Recent studies indicate that mitophagy and healthy mitochondrial function are critical to the survival of stem cells, but how these processes are regulated in MSCs is unknown. Here we show that MSCs manage intracellular oxidative stress by targeting depolarized mitochondria to the plasma membrane via arrestin domain-containing protein 1-mediated microvesicles. The vesicles are then engulfed and re-utilized via a process involving fusion by macrophages, resulting in enhanced bioenergetics. Furthermore, we show that MSCs simultaneously shed micro RNA-containing exosomes that inhibit macrophage activation by suppressing Toll-like receptor signalling, thereby de-sensitizing macrophages to the ingested mitochondria. Collectively, these studies mechanistically link mitophagy and MSC survival with macrophage function, thereby providing a physiologically relevant context for the innate immunomodulatory activity of MSCs.

## Abstract (web summary)

The physiological role of crosstalk between mesenchymal stem cells (MSC) and macrophages is unclear. Here, Phinney *et al*. show that MSCs transfer mitochondria to macrophages under oxidative stress, and desensitize macrophages to mitochondria by using microvesicles to repress Toll receptor signalling.

## Graphical abstract

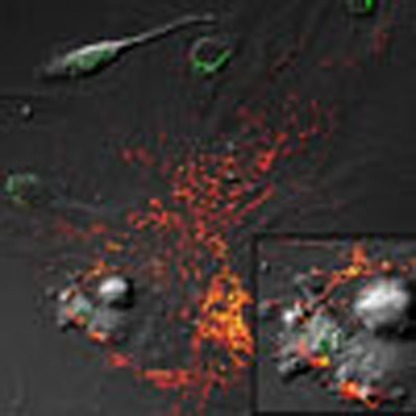

Mesenchymal stem cell (MSC)-based therapies have yielded beneficial effects in a broad range of animal models of disease and several human clinical trials. Nevertheless, their mode of action *in vivo* remains ambiguous. Early studies indicated that MSCs promoted tissue repair via direct differentiation; however, data showing cells that exhibited transient and low engraftment *in vivo* rebutted this hypothesis. It is now believed that MSCs achieve a therapeutic effect *in vivo* via paracrine action^1^,^2^,^3^. This paradigm shift was based initially on studies indicating that conditioned medium from cultured MSCs reproduce some of the beneficial effects of intact cells^4^,^5^. Subsequent studies have identified a long list of paracrine-acting factors secreted by MSCs that contribute to their therapeutic potency^1^,^2^,^3^. More recent studies indicate that the cells also shed extracellular vesicles including exosomes (50–100 nm in diameter) and microvesicles (MVs; 0.1–1 μm in diameter) into the extracellular space^6^,^7^,^8^,^9^,^10^,^11^ and that MSC-derived exosomes protect mice from myocardial or renal ischaemia, and pulmonary arterial hypertension^12^,^13^,^14^,^15^. While the isolation of exosomes requires differential ultracentrifugation, MVs can be isolated from cell culture supernatant by low-speed centrifugation^16^,^17^,^18^,^19^. The role of MVs in MSC biology is largely unknown.

MSCs reside within the bone marrow stem cell niche and regulate haematopoietic stem cell (HSC) maintenance via crosstalk with macrophages^20^,^21^,^22^,^23^,^24^,^25^. The bone marrow niche represents a low-oxygen environment, and changes in oxygen concentration affect MSC and HSC fate^26^,^27^. We recently reported that culture expansion of MSCs in atmospheric oxygen induces mitochondrial oxidative stress (mtROS) that compromises cell growth and survival^28^. However, the programme regulating the quality control of mitochondria in MSC is poorly understood. Mitophagy and allophagy regulate mitochondrial numbers in stem cells and mediate the maternal inheritance of mitochondrial DNA (mtDNA) by facilitating the elimination of paternal mitochondria^29^. Recent studies indicate that mitochondria can be transferred between cells, and cross-talk between MSCs and renal, myocardial and lung epithelial cells involve mitochondrial transfer^30^,^31^,^32^. For example, MSCs introduced into the lungs of lipopolysaccharide (LPS)-treated mice form connexin 43 gap junctional channels and transfer mitochondria to the alveolar epithelium^33^. However, circulation of mitochondria induces inflammatory responses similar to sepsis^34^. These inflammatory responses have been attributed to the release by mitochondria of damage-associated molecular patterns including mtDNA, which stimulate pattern recognition receptors^34^,^35^,^36^. Therefore, while mitochondrial transfer is purported to contribute to the paracrine action of MSCs, its physiological relevance is unclear. Indeed, loss of mitochondria is likely to have detrimental effects on cell survival.

We hypothesized that oxidative stress and mitochondrial dysfunction induce cell survival mechanism in MSCs that include mitochondrial transfer, thereby providing a physiological relevant explanation for this behaviour. To test this hypothesis, we studied mitophagy in MSCs and the role of macrophages in this process since these two cell types are in close proximity within the HSC niche^20^,^24^,^25^. We demonstrate that under standard culture conditions MSCs undergo mitophagy and use arrestin domain-containing protein 1-mediated MVs (ARMMs) to unload mitochondria, which are engulfed by macrophages and re-utilized to increase bioenergetics. Moreover, we show that MSCs tolerize macrophages to mitochondrial transfer by shedding exosomes that modulate Toll-like receptor (TLR) expression and inflammatory signalling via transfer of regulatory microRNAs both *in vitro* and in an *in vivo* model of lung injury.

## Results

### MSCs undergo mitophagy in response to oxidative stress

**Figure 1:**
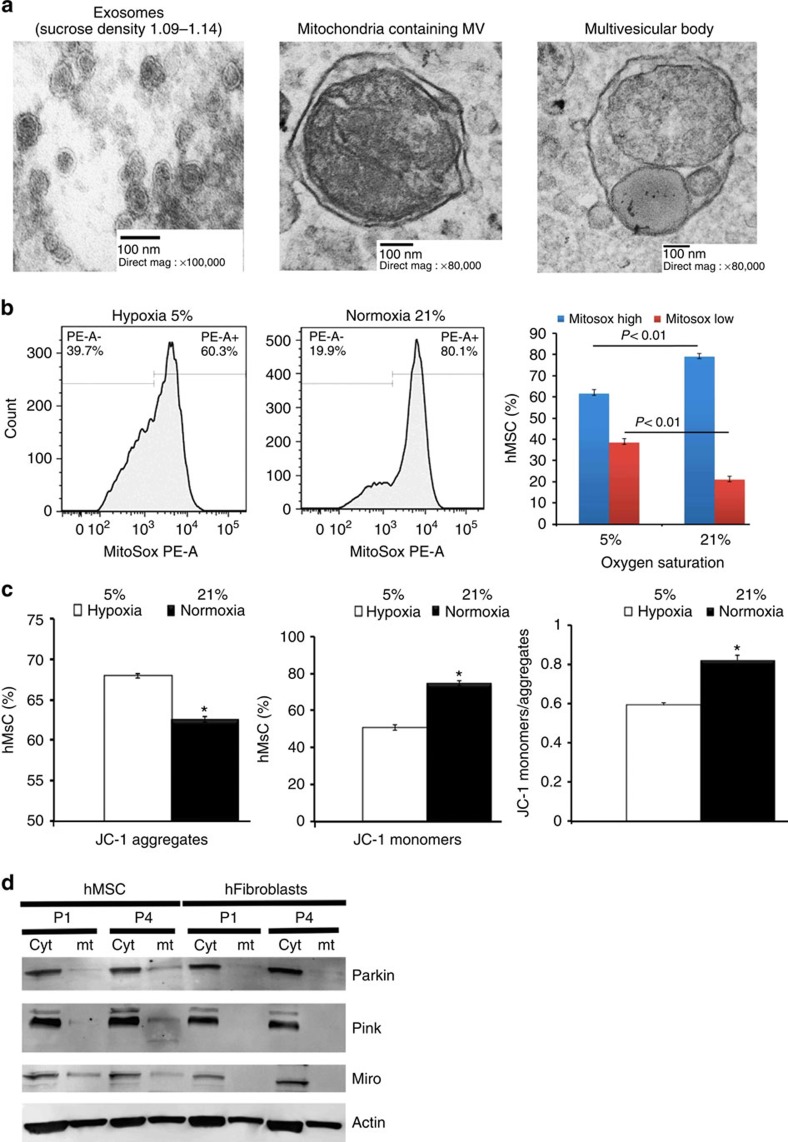
MSC-derived MVs contain depolarized mitochondria. (**a**) Left panel, electron microscopy of vesicles isolated from sucrose densities of 1.11 and 1.14 g ml^−1^ and purified using differential ultracentrifugation (100,000*g* per 18 h) reveal a typical exosome morphology. Middle panel, MVs over 100 nm in size recovered from human MSC-conditioned medium following low-speed (10,000 g per 1 h) centrifugation contain structures conforming to the morphology of mitochondria. Right panel, MVs contain closely packed-vesicles and entire mitochondria (multivesicular body (MVB)) representing autophagosomes. (**b**) Left panel, flow cytometric analysis of MitoSOX Red-stained human MSCs expanded in 5 or 21% oxygen for 7 days. Right panel, quantification of the flow cytometric data. Plotted values (mean±s.e.m.) represent four replicates for each sample using three distinct replicate cultures from each experimental group. (**c**) Mitochondrial membrane potential of human MSCs from **b** determined using JC-1 staining. Expansion in 21% oxygen results in partial depolarization of mitochondria as evidenced by accumulation of JC-1 monomers (*P<0.005, Student’s-*t* test versus MSCs in 5% oxygen). (**d**) Western blot analysis (Supplementary Fig. 1) of cytoplasmic or mitochondrial extracts prepared from human MSCs expanded in 21% oxygen for the indicated passage numbers (P1 or P4) reveals Parkin mitochondrial translocation and Pink1 kinase activation in human MSCs but not human dermal fibroblasts. Data are representative of a single experiment repeated five times.

Human MSCs shed from their surface a diverse subpopulation of vesicles. To characterize these vesicles, we performed electron microscopy on those recovered from MSC-conditioned medium by differential ultracentrifugation (100,000 *g* for 18 h). This analysis demonstrated the presence of 50–100-nm vesicles that are morphologically consistent with exosomes (Fig. 1a, left). Flotation of the 100,000 *g* pellets on sucrose gradients followed by western blot and fluorescent activated cell sorting (FACS) further demonstrated that these vesicles expressed the exosomal markers’ milk fat globule factor 8 (Mfge8) and the tetraspanins CD9 and CD63, respectively (Supplementary Fig. 1A). Centrifugation of conditioned medium at low speeds (10,000 *g*) revealed the presence of larger vesicles (>100 nm) that contain subcellular mitochondrial structures including outer and inner membranes and cristae, and expressed the mitochondria-specific protein ATP synthase as evidenced by immuno-gold labelling (Fig. 1a, centre and Supplementary Fig. 1B). MSCs also release larger multivesicular bodies containing lysosome-like vesicles and entire mitochondria, suggesting that these organelles were selected for mitophagy by targeting to autophagosomes (Fig. 1a, right).

To determine whether culture expansion of MSCs impairs mitochondrial function, we expanded cells under physiological oxygen levels (5% O_2_) or standard culture conditions (21% O_2_) and quantified mtROS levels and mitochondrial membrane potential by staining with MitoSOX Red and JC-1, respectively^37^. FACS analysis of stained cells confirmed that exposure to 21% oxygen resulted in a significant increase in mtROS (Fig. 1b) and a concomitant decrease in mitochondrial membrane potential as demonstrated by the accumulation of JC-1 monomers (Fig. 1c). Moreover, western blot analysis revealed that prolonged exposure to 21% oxygen activated the Pink1/Parkin-mediated pathway of mitophagy in MSCs but not in human fibroblasts cultured under identical conditions (Fig. 1d). Herein, increased mitochondrial expression of Parkin and Pink1 kinase was accompanied by Pink1 kinase activation as evidenced by the presence of lower molecular weight moieties of the protein and reduced Miro levels in mitochondrial extracts from P4 versus P1 MSCs (Fig. 1c and Supplementary Fig. 1C). Importantly, Pink1 targets Miro for degradation, thereby severing the connection of the mitochondria to the cytoskeleton and facilitating its incorporation into the phagosome^38^. Consistent with these results, western blot analysis also revealed that mitochondria-containing MVs expressed microtubule-associated protein 1 light chain 3 (LC3) and autophagy-related protein 12, which are highly enriched in MVs as compared with whole-cell extracts (Supplementary Fig. 1D). Therefore, these MVs are characteristic of autophagosomes^39^.

### MSCs package mitochondria in MVs for cellular transfer

**Figure 2:**
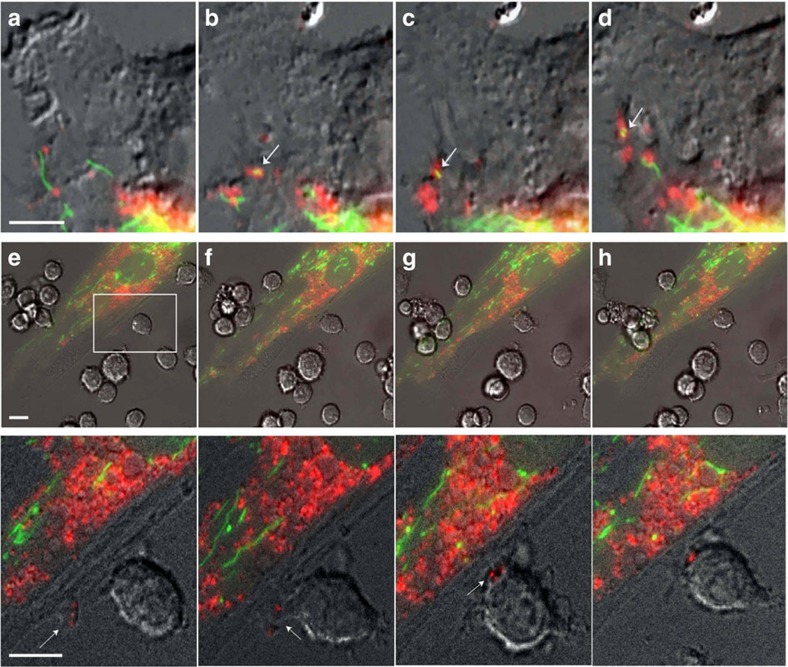
MSC outsource mitophagy to macrophages. (**a**–**d**) Differential interference contrast (DIC) fluorescence overlay of live human MSCs expressing fluorescent proteins that target mitochondria (green) and phagosomes (red) shows mitochondria being loaded into phagosomes (arrows), which are then shuttled to the plasma membrane for extrusion (also see Supplementary Movie 1). (**e**–**h**) Inset shows a representative macrophage interacting with a human MSC. This interaction is shown as a time sequence (5 min intervals) in the lower images and in Supplementary Movie 3. The inset demarcates the area in the human MSC plasma membrane where the membrane blebs outwards and accumulates vesicles. Macrophages nibble the surface of human MSCs and uptake mitochondrial laden phagosomes from blebs budding (arrows) from the plasma membrane of the human MSCs. Scale bars, 10 μ.

To examine mitophagy in MSCs in more detail, MSCs were infected with baculoviruses encoding green fluorescent protein (GFP) fused to the E1alpha pyruvate dehydrogenase leader peptide, which drives transport to the mitochondria, and LC3 fused to a red fluorescent protein (RFP) to allow tracking to the phagophore^39^,^40^. Fluorescent microscopy confirmed that the GFP-labelled mitochondrial network is in close proximity to RFP-LC3-labelled phagosomes (Fig. 2a). Live cell imaging further revealed that mitochondria are loaded in the cytoplasm into LC3-containing vesicles, which migrate towards the cell periphery and are incorporated into outward budding blebs in the plasma membrane (Fig. 2b–d and Supplementary Movie 1). Western blot analysis further revealed that RFP-LC3-MVs also expressed the endosomal sorting complex required for transport (ESCRT)-associated proteins’ tumour suppressor gene 101 (TSG101) and arrestin domain-containing protein 1 (ARRDC1)^11^,^41^ (Supplementary Fig. 1D). Collectively, these results indicate that MSCs employ the release of ARMMs to extrude mitochondria at their cell surface. Moreover, MSCs exhibited marked increases in apoptosis when treated with Bafilomycin A1 or low concentrations (3–5 μM) of chloroquine, which block the mitophagy flux, indicating that this process is critical for MSC survival (Supplementary Movie 2).

**Figure 3:**
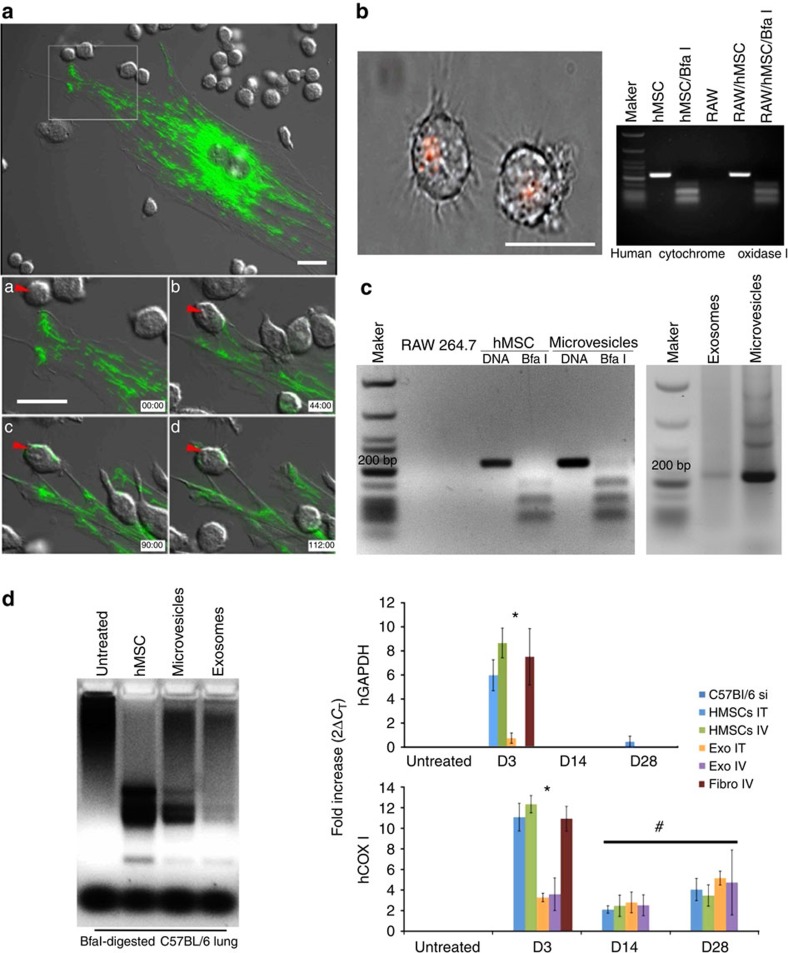
MSC transfer mitochondria to macrophages and lung tissues. (**a**) Top panel is DIC fluorescent overlay at time 0 of primary human MSCs infected with Organelle Lights to label mitochondria (green) and co-cultured with mouse (RAW 264.7) macrophages. Lower panels, time sequence at 45 min intervals showing transfer of green-labelled mitochondria from the a MSC to a macrophage (red arrow, see Supplementary Movie 4 for transfer of mitochondria in filamentous form, and Supplementary Movie 5 in which GFP signal is compensated to allow the tracking of the transferred mitochondria into macrophages). (**b**) Left panel, photomicrograph of FACS-sorted mouse macrophages that were co-cultured with mitochondria-labelled (RFP) human MSCs clearly show retention of RFP label. Right panel, electrophoretic pattern of human COX I PCR product treated with or without Bfa1 after amplification from the indicated cell sources. (**c**) MSC-derived exosomes and MVs express the Bfa1-sensitive 228-bp COX I mtDNA PCR product detected in human MSCs (**b**). (**d**) Left panel, electrophoretic pattern of Bfa1-digested human COX1 PCR product amplified from mouse lung DNA isolated 14 days after the intravenous administration of human MSCs, human MSC-derived MVs or exosomes. Right panel, human GAPDH and human COX1 relative expression levels quantified by RT–PCR in mouse lung (3–28 days) after a single (intratracheal (IT) or intravenous (IV)) injection of human MSCs, human MSC-derived exosomes or human fibroblasts. *P<0.001, #*P*<0.001 by ANOVA compared with untreated mouse lung. Plotted values (mean±s.e.m.) are from experiments repeated four times. Scale bars, 20 μ.

Next, we co-cultured GFP-labelled human MSCs from above with primary human or mouse macrophages. Live cell imaging revealed that macrophages nibble the plasma membrane of MSCs, establishing cell contact at areas where membrane blebs are enriched in RFP-labelled vesicles, which are subsequently stripped by the macrophage (Fig. 2e–h and Supplementary Movie 3). This activity was also observed between mouse macrophages and primary human MSCs (Fig. 3a and Supplementary Movies 4 and 5) but was not evident when macrophages were co-cultured with mouse or human fibroblasts (Supplementary Fig. 2). In a subsequent experiment, we co-cultured the macrophage cell line RAW 264.7 with human MSCs containing RFP-labelled mitochondria (10:1 ratio) for 4 h and recovered macrophages using FACS after staining with antibodies that recognize macrophage epitopes (that is, F4/80) not expressed by MSCs. Sorted macrophages were cultured for up to 2 weeks in RPMI media, which do not support MSC expansion and survival. Fluorescent microscopy of these macrophages revealed clear evidence of cell-associated RFP derived from human MSCs (Fig. 3b). To confirm these findings, we demonstrated using PCR amplification that these macrophages expressed the mitochondrial specific transcript human cytochrome *c* oxidase I (MT-COX I), which was confirmed on the basis of the restriction fragment pattern obtained after digestion of the PCR product with Bfa1 (Fig. 3b). This PCR product was not detected in mouse macrophages because of limited sequence homology between the two genes^30^ but was detected in human MSC-derived MVs as expected (Fig. 3c and Supplementary Fig. 3A). Lastly, we co-cultured Cy5-labelled human MSCs with macrophages that were pre-incubated with or without dextran sulfate (100 μg ml^−1^), a nonspecific inhibitor of phagocytosis. Live cell imaging showed phagocytosis of MVs by macrophages over a period of 18 min, and confocal microscopy confirmed that the engulfed Cy5-labelled vesicles resided within the cell body of the macrophage (Supplementary Movie 6). However, MV uptake was blocked in macrophages pre-treated with dextran sulfate as evidenced by the accumulation of Cy5-labelled MVs on the macrophage surface (Supplementary Movie 7).

To track the *in vivo* transfer of mitochondria, we systemically administered RFP-labelled human MSCs into C57BL/6 mice expressing a GFP reporter under control of the endothelial specific Tie2 promoter. At 24 h post injection, GFP-labelled endothelial cells, epithelial cells and macrophages that contained RFP-labelled mitochondria were visible (Supplementary Fig. 3B). Bfa1 digestion of mouse lung DNA following intravenous administration of human MSCs, exosomes or MVs yielded a pattern of restriction similar to those observed in RAW 264.7 macrophages (Fig. 3d). To follow the fate of viable human MSCs in the mouse lung, we measured the abundance of human-specific GAPDH transcripts via reverse transcriptase–PCR (RT–PCR)^42^. Human GAPDH mRNA was not detected in the lung tissue of untreated mice but was detected at 3 days post injection of human MSCs or human fibroblasts (Fig. 3d). However, expression rapidly declined and was no longer evident by 14 or 28 days post transplant, consistent with the clearance rate of cells from lung tissue. Expression of human COXI mRNA in mouse lung mirrored that of human GAPDH following injection of human fibroblasts and was detected at 3 days but not 14 or 28 days post transplant. In contrast, human COXI transcripts were detected up to 28 days post injection of human MSCs, indicating that mouse lung tissue retained mtDNA long after the disappearance of viable human MSCs (Fig. 3d). Thus, MSC-derived vesicles constitute an effective mechanism to transfer mtDNA into the mouse lung.

### MSC extracellular vesicles enhance macrophage energetics

**Figure 4:**
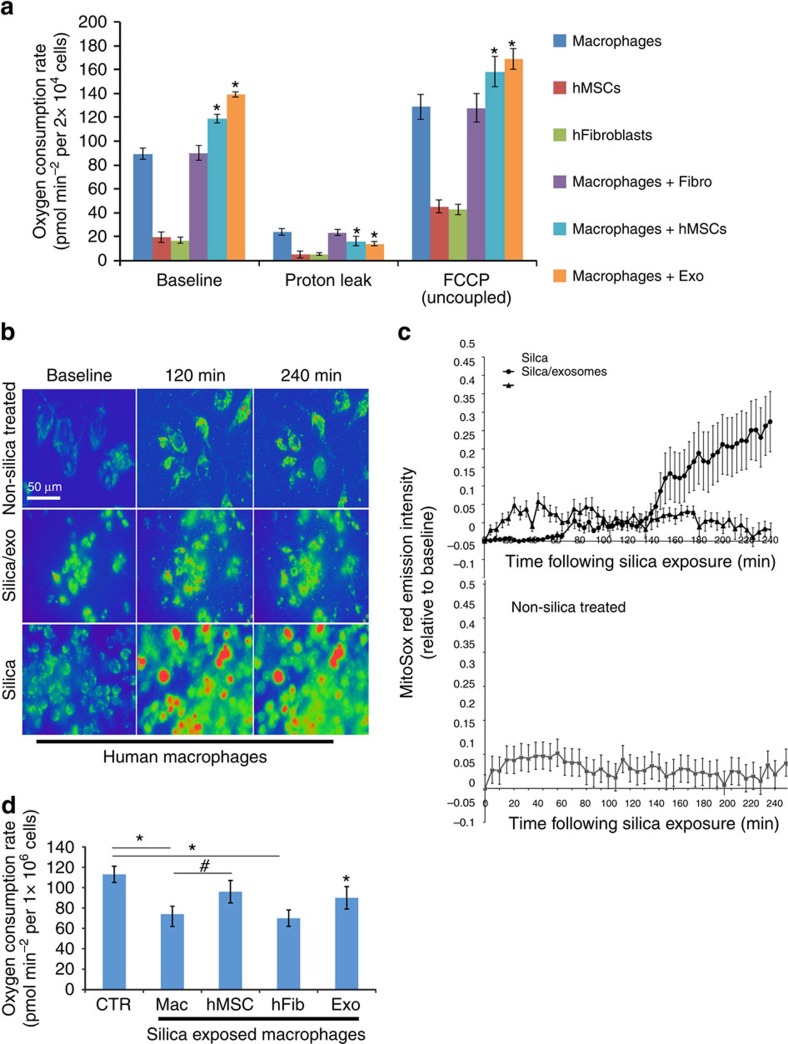
MSCs enhance macrophage bioenergetics. (**a**) Mitochondrial respiration of human macrophages, human MSCs or human fibroblasts was measured as OCR using the XF technology. Macrophages were co-cultured with or without human MSCs or fibroblasts (1:10 ratio) or treated with human MSC-derived exosomes (40 μg per protein) in the presence or absence of Oligomycin A and FCCP to differentiate ATP-linked respiration from the proton leak. Plotted data (mean±s.e.m.) were performed using six replicates per sample and repeated three times. (**b**) Pseudocoloured photomicrographs (0–240 min) of MitoSOX Red-stained macrophages that were non-stimulated (upper panel), or treated with silica (20 μg cm^−2^, lower panel) or silica plus human MSC-derived exosomes (added 10 min after silica, middle panel). Scale bars, 50 μ. (**c**) Time course of MitoSOX Red emission by human macrophages treated as in **b**. Figure is representative of five exposures (nine stages positions per test and 6 cells per stage). (**d**) OCR as in **a** of silica-exposed macrophages treated with or without human MSCs, human MSC-derived exosomes or human fibroblasts. Plotted values (mean±s.e.m.) are from experiments repeated three times, *P<0.05 as compared to control, ^#^*P*<0.05 as compared to silica treated macrophages, as determined by Student's *t*-test.

To study the effect of MVs on macrophage bioenergetics, we analysed oxygen consumption rates (OCRs) using the SeaHorse technology. Human macrophages exhibit higher basal OCR than human MSCs or human fibroblasts (Fig. 4a). Co-culture of macrophages with human MSCs (Mac+hMSC) or MSC-derived exosomes (Mac+Exo) but not human fibroblasts (Mac+Fibro) significantly (analysis of variance (ANOVA) followed by Student–Neuman Keuls (SNK) *post-hoc* pairwise comparisons) increased their OCR, suggesting that MSCs or MSC-derived exosomes alter macrophage bioenergetics (Fig. 4a). Next, we repeated these measurements after treatment of cells with oligomycin A, an inhibitor of ATP synthase, which is required for the oxidative phosphorylation of ADP to ATP. These conditions differentiate ATP-linked respiration from the proton leak. Macrophages exhibited a higher level of proton leak as compared with human MSCs and fibroblasts, and proton leak was significantly (ANOVA followed by SNK *post hoc* pairwise comparisons) reduced following co-culture with human Mac+Exo but not Mac+Fibro (Fig. 4a). Co-culture with human MSCs (Mac+hMSC) also significantly (ANOVA followed by SNK *post hoc* pairwise comparisons) reduced proton leak in macrophages. We also repeated the OCR measurements following treatment of cells with the uncoupling agent carbonyl cyanide 4-(trifluoromethoxy) phenylhydraone (FCCP) to determine how cells respond to an increase in ATP demand. All three cell types responded to FCCP treatment with increased OCR, and the magnitude of the response was greater in macrophages as compared with human MSCs and fibroblasts. Moreover, OCR was significantly increased in FCCP-treated macrophages following co-culture with human MSCs (Mac+hMSC) or human Mac+Exo but not Mac+Fibro (Fig. 4a).

**Figure 5:**
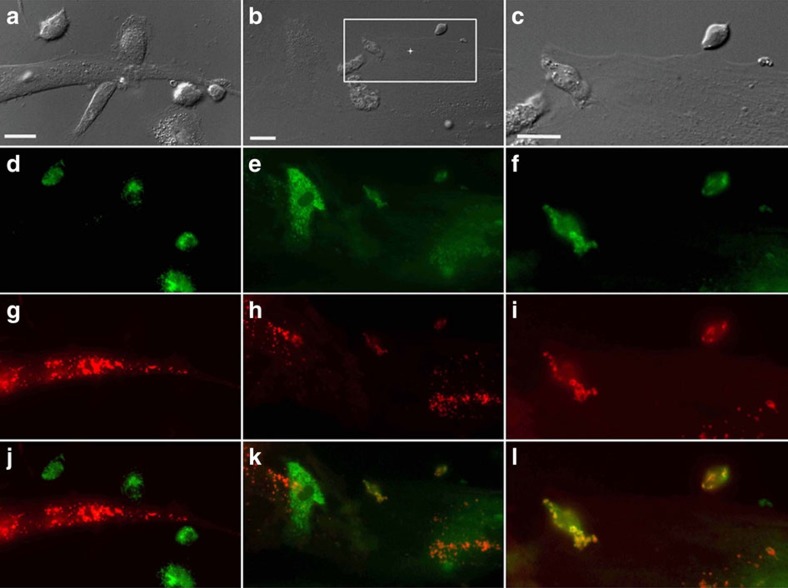
Mitochondrial transfer from human MSCs is followed by fusion inside macrophages. Human MSCs and human macrophages (1 × 10^5^) were infected separately with Organelle Lights to label human MSC mitochondria (red) and macrophage mitochondria (green). Twenty-four hours following infection, macrophages were harvested and co-incubated with the human MSCs for 2 h. Images were collected using an inverted Nikon TiE fluorescent microscope equipped with a × 60 oil immersion optic and NIS Elements Software. Organelle Lights were excited using a Lumencor diode-pumped light engine and detected using an ORCA-Flash4.0 sCMOS camera. (**a**,**b**) DIC images of two separate fields within the same dish. (**c**) A zoomed image of the outlined section within **b** (scale bars, 20 μ). The fluorescence-based images for each field appear in the panels below the DIC images, with **d**–**f** showing macrophage mitochondria (green); **g**–**i** showing human MSC mitochondria (red); and **j**–**l** showing the overlay with yellow indicative of colocalization of human MSC and macrophage mitochondria. Not every macrophage was shown to take up human MSC mitochondria (**a**,**d**,**g**, **j**).

To examine the effect of MSC or exosomes on macrophage bioenergetics under conditions of altered homeostasis, we exposed macrophages to silica particles. Silica exposure results in a burst of mtROS production as evidenced by changes in MitoSOX Red fluorescence intensity; however, this effect is largely mitigated in macrophages incubated with human MSC-derived exosomes (Fig. 4b,c). Silica exposure also decreased macrophage OCR, but this decrease was reversed by co-culture with human MSCs or human MSC-derived exosomes but not with human fibroblasts (Fig. 4d). The fact that transfer of partially depolarized mitochondria from MSCs to macrophages enhances that macrophage bioenergetics appears paradoxical. However, loss of mitochondrial membrane potential as a result of MSC expansion is not absolute as mitochondria exhibit residual membrane potential as evidenced by the concentration of JC-1 aggregates (Fig. 1c). This indicates that the mitochondrial membrane is not collapsed and the mitochondria are still capable of undergoing fusion. To determine whether these mitochondria are recycled in macrophages by fusion, we co-cultured human MSCs with macrophages after labelling cells with two different MitoTracker dyes (Red and Green)^43^. Live cell imaging clearly demonstrated the transfer and subsequent fusion (yellow colour in merged images) of RFP-labelled, human MSC-derived mitochondria with GFP-labelled mitochondria within human macrophages (Fig. 5). These data indicate that under oxidative stress MSCs outsource mitophagy to macrophages to unload partially depolarized mitochondria, which are recycled via fusion by macrophages thereby enhancing their bioenergetics.

### MSC-derived exosomes are enriched in microRNAs

**Figure 6:**
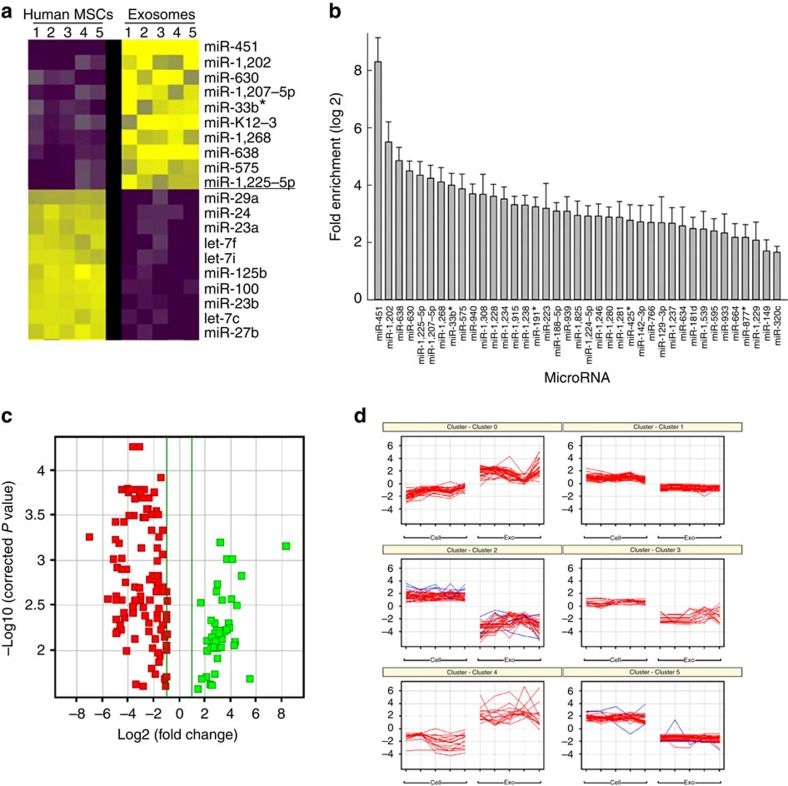
RNA expression profile in human MSCs and their exosomes. (**a**) Heatmap illustrating the 10 microRNAs most highly enriched in human MSCs versus their corresponding exosomes. Every row represents a microRNA and every column a cell or exosome, and yellow and purple represent increased or decreased expression, respectively. (**b**) Plotted values represent the means log 2 fold enrichment of exosomal versus human MSC microRNAs (*n*=5 microarrays of different MSCs cell lines; *P*<0.05, ANOVA followed by Holm–Sidak *post hoc* pairwise comparisons). (**c**) Data in **b** show distribution of differentially expressed microRNAs between samples based on the –log base 10 significant *P* value (<0.05) and with a relative fold change of >2 (in log base 2). Green and red squares represent increasingly and decreasingly expressed microRNAs, respectively, in exosomes versus human MSCs. (**d**) Computational analysis of human MSCs and exosomes from five donors demonstrates that microRNAs isolated in exosomes cluster among different donors.

Exosomes transfer RNAs between cells^8^. We hypothesized that this process may be exploited by MSCs to tolerize macrophages against mitochondrial transfer. To explore this possibility, we analysed the RNA content of human MSC-derived exosomes. Using microRNA microarray analysis, we identified 156 (45 increased; 111 decreased) microRNAs that differed (log2>1.0, *P*<0.05 (ANOVA followed by Holm–Sidak *post hoc* pairwise comparisons) in abundance between exosomes compared with their parent MSCs. The 10 microRNAs that exhibited the greatest increase included miR451a (316-fold), miR1202 (45-fold), miR630 (40-fold) and miR638 (28-fold), while microRNAs that exhibited the greatest decrease in exosomes and were enriched in MSCs included miR125b (148-fold) and miR21 (91-fold; Fig. 6a,b). This pattern of microRNA expression was conserved in MSC-derived exosomes obtained from five human donors (Fig. 6c,d).

### MSC-derived exosomes inhibit TLR signalling in macrophages

**Figure 7:**
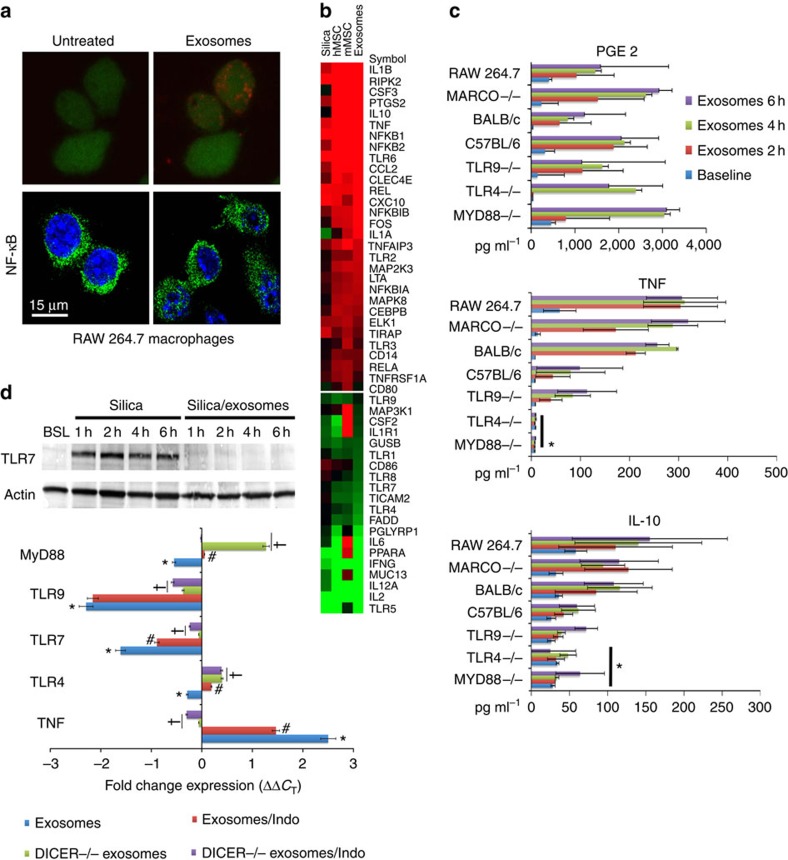
MSC-derived MVs inhibit TLR signalling in macrophages. (**a**) Upper panels, confocal microscopy showing intracellular localization of Cy5-labelled exosomes within macrophages 18 min post administration. Lower panel, nuclear localization of NF-κB in macrophages 2 h post administration of exosomes. Scale bars, 15 μ. (**b**) Partial heatmap illustrating mRNA levels of 84 TLR-associated transcripts in macrophages at 8 h post treatment with silica (20 mg cm^−2^), human or mouse MSCs (1:10 ratio) or human MSC-derived exosomes (40 μg protein). Transcript order is highest (top) to lowest (bottom), and each row represents a gene and each column a specific treatment. Red and green illustrates increased or decreased gene expression, respectively. Experiments were repeated four times. (**c**) Effect of exosome treatment on PGE2, TNF and IL-10 secretion in macrophages from the indicated mouse strains. Plotted data (mean±s.e.m.) were from experiments repeated five times. *P<0.05 compared with C57BL/6J or BALB/CJ macrophages as determined by ANOVA). (**d**) Upper panel, western blot illustrating the time-dependent effect of silica or human MSC-derived exosomes on expression of TLR7 in macrophages. Lower panel, fold change in expression of the indicated transcripts in macrophages RT–PCR demonstrating the negative regulation of exosomes on macrophage expression of TLR genes. Plotted data (mean±s.e.m.) are from experiments repeated four times. *P<0.05 compared with baseline. #*P*<0.05 compared with native exosomes by Student’ *t*-test. †*P*<0.05 compared with the effect of native exosomes and indomethacin (Indo) treated by Student’s *t*-test.

Mitochondrial uptake can induce inflammation via activation of pattern recognition receptors^34^. Therefore, given the presence of mtDNA and microRNAs in MSC-derived MVs and exosomes, respectively, we hypothesized that exposure to these vesicles would tolerize macrophages to mitochondrial transfer by inducing changes in TLR expression. Subsequently, we profiled the expression of 84 TLR-associated transcripts in mouse macrophages. We contrasted these results with those observed in macrophages that were co-cultured with mouse or human MSCs, human MSC-derived exosomes or silica particles, which when phagocytized induce macrophage activation^44^. Co-culture of macrophages with MSC-derived exosomes induced nuclear translocation of the transcription factor NF-κB (Fig. 7a) resulting in significant changes (>2.5-fold increase or decrease) in expression of 50 of the 84 TLR-associated transcripts (Fig. 7b). For example, compared with silica-exposed macrophages those treated with exosomes exhibited significant (>2.5-fold) increases in transcripts associated with cytokine signalling including interleukin (IL)-1β, prostaglandin endoperoxide synthase 2 (PTGS2, aka COX2), granulocyte colony-stimulating factor 3 (CSF3), IL-10, chemokine (C–C motif) ligand 2 (CCL2, aka MCP-1), NF-κB-chemokine (C–X–C motif) ligand 10 (CXCL10), tumour necrosis factor (TNF) and reticuloendotheliosis oncogene (Rel; Fig. 7b). In contrast, transcripts encoding proteins involved in MyD88-dependent signalling (MyD88, TLR 1,4,5,7,8 and 9, IRAK1 and TRAF6), TRIF-dependent signalling (TLR adaptor molecule 1 (TICAM1) and TICAM2) and TLR-related signalling (CD80, CD86, IL-2, IL-12, Interferon gamma, PGLYRP1 and CSF2) were downregulated.

MSCs secrete PGE2 that acts on prostaglandin receptors of LPS-stimulated macrophages to enhance their production of the anti-inflammatory cytokine IL-10 (ref. 45). However, this effect of MSCs was abrogated in macrophages from TLR4, MyD88, TNFR1 or COX2-deficient mice^45^. Consistent with these results, exosome treatment of non-stimulated macrophages augmented secretion of PGE2, TNF, IL-10 and IL-1-receptor antagonist (Fig. 7c), which may reprogramme macrophages^3^. These responses recapitulate those observed when macrophages are exposed to intact human or mouse MSCs, except that IL-6, CSF2 and IL-1 receptor 1 were increased more following exposure to mouse MSCs (Fig. 7b).

Subsequently, we treated TLR-signalling-deficient macrophages (TLR4^−/−^, TLR9^−/−^, MyD88^−/−^) or scavenger receptor-deficient macrophages (MARCO^−/−^) with MSC-derived exosomes. As shown in Fig. 7c, PGE2 production was similar following exosome treatment in all signalling-deficient macrophages as compared with wild-type cells from strain-matched C57BL/6J or BALB/CJ mice. In contrast, secretion of TNF and IL-10 was significantly (ANOVA followed by SNK *post hoc* pairwise comparisons) reduced in TLR4^−/−^ and MYD88^−/−^ macrophages as compared with wild-type cells following exosome treatment, and IL-10 secretion was also significantly reduced in macrophages from TLR9^−/−^ mice (Fig. 7c). These data confirm the importance of TLRs and in particular MyD88-dependent pathways in mediating exosome-induced effects on macrophage function. Lastly, we showed that pre-incubation with dextran sulfate significantly (ANOVA followed by SNK *post hoc* pairwise comparisons) reduced the release of PGE2, TNF and IL-10 by exosome-treated macrophages, confirming the need for phagocytosis of MSC-derived vesicles in this process (Supplementary Fig. 4A).

To examine the role of microRNAs in macrophage tolerization, we treated RAW 264.7 cells, which use TLRs to recruit autophagy proteins in phagosomes to degrade its cargo^46^, with exosomes derived from human MSCs transfected with or without an short-hairpin RNA (shRNA) designed to inhibit DICER expression in the presence or absence of indomethacin, a cyclooxygenase inhibitor (Supplementary Fig. 4B). Treatment of naive RAW 264.7 macrophages with native exosomes enhanced TNF and reduced TLRs and MyD88 mRNA expression over 24 h (Fig. 7c), while treatment of silica-exposed macrophages with exosomes ameliorated TLR7 induction following silica exposure (Fig. 7c). Pre-incubation of RAW 264.7 macrophages with indomethacin before treatment with native exosomes, or treatment with exosomes from DICER knockout MSCs significantly (ANOVA followed by SNK *post hoc* pairwise comparisons) reduced the observed effects on TLR mRNA expression (Fig. 7c) and reduced secretion of proteins such as TNF, MIP, MCP1, KC and IP-10 associated with macrophage activation (Supplementary Fig. 4B,C). The inhibitory effects of indomethacin were restricted to TLR4 and MyD88 mRNA, while the effects of DICER-deficient exosomes were of greater magnitude and also involved negative regulation of TLR 7 and 9 (Fig. 7c). Concomitant treatment with indomethacin and exosomes from DICER-deficient exosomes demonstrated additive effects (Fig. 7c).

Importantly, miR-451 is one of the most abundantly expressed microRNAs in MSC-derived exosomes, but its maturation occurs independent of DICER^47^. Therefore, its expression is not altered in exosomes from DICER knockdown MSCs. MiR-451 negatively regulates cytokine production in dendritic cells infected with influenza virus^48^. Consistent with these results, transfection of RAW 264.7 macrophages with a miR-451 mimic significantly (Student’s *t*-test) decreased TNF mRNA expression in non-stimulated macrophages, and inhibited mRNA expression and protein release in silica-exposed macrophages (Supplementary Fig. 4D). In contrast, treatment of cells with a miR-451 antagomir yielded the opposite result. These data confirm a role of exosome-derived microRNAs in regulating cytokine expression in macrophages.

### MSC exosomes attenuate monocyte activation and silicosis

**Figure 8:**
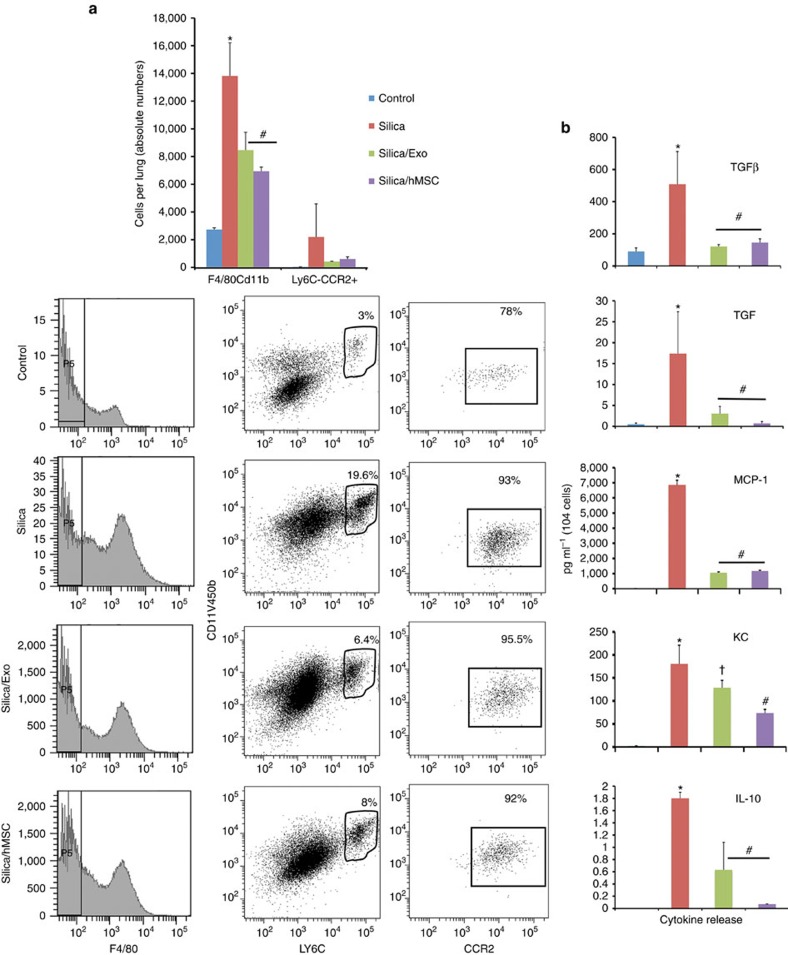
Human MSCs and their exosomes prevent the accumulation of Ly6C^hi^ monocytes in the lungs of silica-exposed mice. (**a**) Upper panel, absolute number of F4/80/CD11b- and Ly6C/CCR2-expressing cells in lung tissue of mice 72 h post administration of saline (50 μl), silica (0.2 g kg^−1^) or silica plus human MSC-derived exosomes (∼3 × 10^11^ exosomes containing 40 μg protein). *P<0.05 compared with saline by *t*-test). Lower panel, representative histograms of flow cytometric data analysed in **a** showing the phenotype and frequency of cells recovered from lung tissue after enzymatic digestion. (**b**) Mulitplex ELISA of inflammatory (TNF, MCP1 and KC) and fibrotic (TGFβ and IL-10) mediators secreted by cultured F4/80/CD11b/ and Ly6C/CCR2 cells from **a**. Plotted values (mean±s.e.m.) are from experiments using *N*=5 animals per group and repeated three times. *P<0.05 compared with saline, #*P*<0.001 compared with silica-treated monocytes by ANOVA.

Circulating MVs enter the bone marrow and reprogramme cells to express proteins of the tissue of vesicle origin^49^. Ly6C^hi^ monocytes are recruited from the bone marrow into the lung in response to injury and play an important role in the pathogenesis of lung fibrosis^50^,^51^. Therefore, we investigated whether MSC or their exosomes are capable of altering the lung recruitment and cytokine production of Ly6C^hi^ monocytes in mice following silica exposure. As shown in Fig. 8a, FACS identified a limited number of Ly6C^hi^ monocytes in the normal mouse lung, which was significantly (ANOVA followed by SNK *post hoc* pairwise comparisons) increased by 72 h post exposure to silica. Moreover, high expression of CCR2 and release of inflammatory (TNF, CCL2 and CXCL1) and fibrotic (transforming growth factor β (TGFβ) and IL-10) mediators indicate that these monocytes are activated (Fig. 8b). In contrast, intravenous administration of human MSCs (500,000 cells) or freshly isolated human MSC-derived exosomes (∼3 × 10^11^ exosomes containing 40 μg protein) at 24 h post-silica exposure (0.2 g·kg^−1^) significantly reduced the extent of Ly6C^hi^ monocyte infiltration into the lung and secreted levels of inflammatory mediators (Fig. 8a,b).

**Figure 9:**
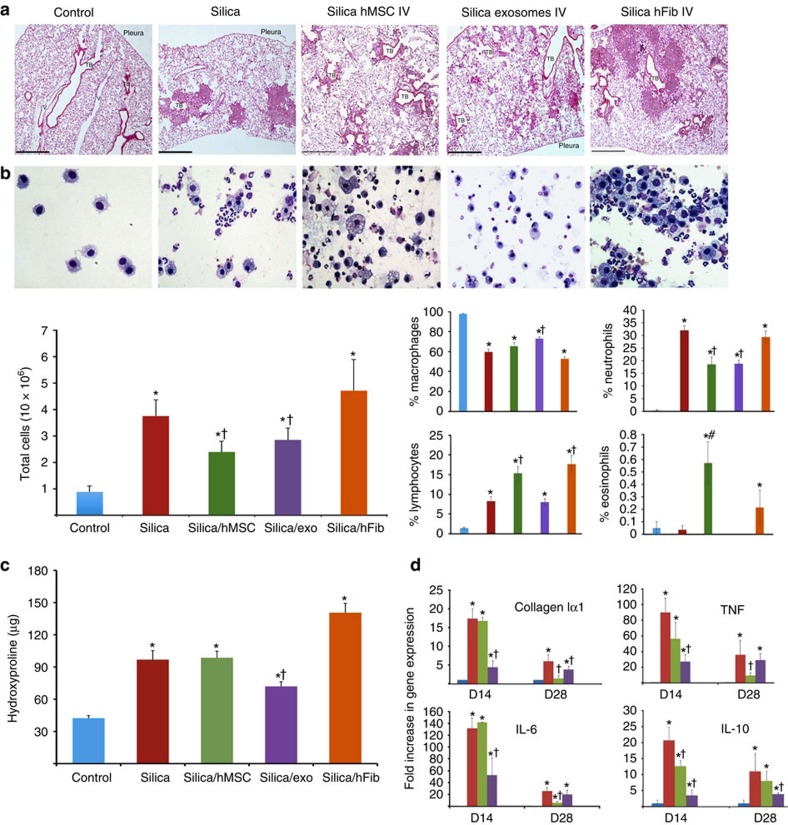
Human MSCs and their exosomes ameliorate experimental silicosis. (**a**) Photomicrographs of lung sections stained with haematoxylin and eosine from mice 28 days after intratracheal administration of silica (0.2 g kg^−1^) alone or followed 3 days later with an intravenous injection of human MSCs, human MSC-derived exosomes (∼3 × 10^11^ exosomes containing 40 μg protein) or human fibroblasts (scale bars, 500 μ). (**b**) Upper panel, photomicrographs of Diff-Quick-stained cytospins of BAL from mice in **a**. Lower panel, differential cell counts showing counts of total cells (left) and percentage of macrophages, lymphocytes, neutrophils and eosinophils (right panels). *P<0.05 compared with control, †*P*<0.05 compared with fibroblasts treated mice by Student’s *t*-test. (**c**) Hydroxyproline content of lung tissue from animals treated as in **a**. *P<0.001 compared with saline by Student’s *t*-test. †*P*<0.05 compared with silica, human MSC or fibroblast by ANOVA. (**d**) Quantification of mouse TNF, IL-6, IL-10 and Col1α1 levels in lung tissue from mice in **a** at 14 and 28 d post treatment. Plotted values (mean±s.e.m.) are representative of experiments using 15 animals per group and repeated three times. *P<0.001 compared with saline by Student’s *t*-test, †*P*<0.05 compared with silica by ANOVA.

Silica induces inflammation and collagen deposition in peri-bronchiolar, silicotic nodule and peri-vascular regions of the lung (Fig. 9a). These lesions are associated with enhanced numbers of inflammatory cells (although the percentage of macrophages decreases, there is an increase in neutrophils and lymphocytes) in bronchoalveolar lavage fluid (BALF, Fig. 9b), significant deposition of lung collagen as measured by hydroxyproline (Fig. 9c) and enhanced expression of pro-inflammatory cytokines (TNF and IL-6) and fibrotic mediators (IL-10 and α(I) collagen) by 14 and 28 days after silica exposure (Fig. 9d). Intravenous administration of human MSCs or exosomes 3 days after silica exposure reduced the size of the silicotic nodules (Fig. 9a), the total number of white blood cells in BALF (Fig. 9b) and expression of inflammatory and pro-fibrotic genes in the lung (Fig. 9d). Administration of exosomes significantly (ANOVA followed by SNK *post hoc* pairwise comparisons) reduced the accumulation of neutrophils and lymphocytes in BALF, while MSCs only reduced the accumulation of neutrophils and induced a slight (<1%) increase in eosinophil count (Fig. 9b). In contrast, the intravenous administration of human fibroblasts significantly exacerbated the inflammatory and fibrotic responses to silica (Fig. 9a–c). Exosomes, but not MSC or fibroblast administration, reduced the accumulation of hydroxyproline in lung tissue 28 days after silica (Fig. 9c).

## Discussion

MSCs modulate macrophage function by a variety of mechanisms, and this crosstalk contributes to their anti-inflammatory activity but the physiological relevance of this crosstalk remains obscure particularly as it relates to the survival and function of MSCs. In this study, we report that during their *ex vivo* culture MSCs transfer partially depolarized mitochondria to macrophages as a pro-survival mechanism in response to oxidative stress and that these mitochondria are repurposed via a process involving fusion to increase macrophage bioenergetics. Moreover, we show that MSCs also desensitize macrophages to mitochondrial transfers by repressing TLR-signalling. Our data indicate that MSCs employ two different types of MVs to achieve these goals. MSCs load mitochondria in the cytoplasm into LC3 containing MVs that are recovered from cell culture media with low-speed centrifugation. These MVs express the ESCRT-I-associated proteins TSG101 and ARRDC1 and are extruded from cells in ARMMs^11^, which bud outwards directly from the plasma membrane where they are identified by macrophages. MSCs also shed exosomes that modulate TLR signalling and cytokine secretion in macrophages, in part, by transfer of regulatory microRNAs.

Previous reports indicate that mitochondria transferred by MSCs improve the energetic activity of the alveolar epithelium of LPS-treated mice^30^,^31^,^32^,^33^ and animal models of rotenone-induced airway injury^52^. However, the beneficial effects of mitochondria were limited to acceptor cells almost completely deficient of mitochondrial function^30^,^53^. Therefore, it is unclear whether rescue is because of the transfer of mitochondria, mtDNA or release of other mediators by MSCs^33^. Importantly, the bone marrow niche contains few, if any, epithelium, so the physiological relevance of this is unclear.

Our data suggest that mitochondrial transfer by MSCs is not altruistic but rather may serve to enhance MSCs’ cell survival by unloading partially depolarized mitochondria. Elimination of depolarized mitochondria is a priority for MSCs that experience high mtROS generation when cultured under atmospheric oxygen tension^28^ since inhibitors of the mitophagy flux induce MSC apoptosis. Unexpectedly, MV-mediated mitochondrial transfer augments macrophage function by improving mitochondrial bioenergetics. As reported for the alveolar epithelial cells, recovery of the energetic function of macrophages is characterized by an increased ability to generate ATP under conditions in which the cells exhibit mitochondrial uncoupling or an enhanced proton leak, and involves protection of the macrophage by reducing mtROS generation. This outcome is consistent with data indicating that transfer of mitochondria, even if partially depolarized, is followed by fusion inside the acceptor macrophage. Notably, several studies have reported that transfer of only a few mitochondria is sufficient to rescue cells depleted of mtDNA by culture in ethidium bromide^30^,^54^,^55^. Furthermore, the current study confirms evidence that exosomes, which do not carry mitochondria, contain nucleic acids^56^, including mtDNA that can be transferred, long term, *in vivo* to the lung. Presence of mtDNA inside exosomes is not surprising as mtDNAs are dispersed throughout the mitochondrial network as histone-free nucleoids with an average size in mammals under 100 nm, and contain a single copy of mtDNA per nucleoid^57^. However, we cannot completely exclude the possibility that the exosome preparations could be contaminated by apoptotic bodies.

Accumulation of mtDNA that escapes mitophagy induces TLR 9-mediated inflammation that in the case of cardiac muscle is associated with heart failure^35^ and mice transplanted with cells harbouring allogeneic mtDNA trigger MyD88 responses to reject these cells^36^. Therefore, silencing TLR responses in macrophages is likely necessary to induce tolerance to transferred mitochondria. Consistent with this hypothesis, we demonstrate that uptake of MSC-derived exosomes represses TLR signalling in macrophages and the production of inflammatory mediators by targeting pathways (TLRs and NF-κB) central to inflammation.

Interestingly, microRNAs present in MSC-derived exosomes are highly conserved between human MSC donors. One such microRNA, miR-451, is highly abundant in exosomes but is expressed at low levels in macrophages and dendritic cells where it regulates cytokine production^58^,^59^,^60^. Mir-451 is known to suppress TNF, and macrophage migration inhibitory factor, which inhibits the anti-inflammatory effects of glucocorticoids and negatively regulates p38 MAPK signalling to protect from diabetic nephropathy^48^,^58^,^59^,^60^. Indeed, ectopic expression of a mir-451 mimic in macrophages inhibits TNF secretion in response to silica. Consistent with these findings, MSC-derived exosomes prevent the recruitment of Ly6C^hi^ monocytes and reduces secretion of pro-fibrotic IL-10 and TGFβ by these cells in the lung of silica-exposed mice. Therefore, these data suggest that, as tested *in vitro*, immunomodulatory activities may have evolved, in part, as a mechanism by which MSCs survive oxidative stress and serendipitously confers on cells the ability to suppress inflammation, in lung injury models. Indeed, our data illustrate a physiological role for the innate immune regulatory activity of MSCs, and in doing so further highlights the important association between MSCs and macrophages *in vivo*.

## Methods

### Human MSC and cell lines

Human MSCs were harvested from small volume aspirates of the iliac crest bone marrow from five healthy adult volunteers by the Center for Preparation and Distribution of Adult Stem Cells formerly at the Tulane University School of Medicine (New Orleans, LA). The Institutional Review Board at the Tulane University approved the procedures involved in the procurement of these cells as previously described^61^. Human MSCs were also provided by the National Heart Lung and Blood Institute (NHLBI)-sponsored Production Assistance for Cellular Therapies Program at the University of Minnesota. Adult normal human dermal or lung fibroblasts were commercially obtained from Lonza (Walkersville, MD). Mouse MSCs were isolated from FVB/NJ or C57BL/6J mice (The Jackson Laboratory) as previously described^28^. All studies employing human or mouse MSCs were performed using low-passage (1–4) populations. Primary mouse macrophages were isolated from the femurs of C57BL/6J, BALB/CJ (The Jackson Laboratory), TLR4−/− (The Jackson Laboratory), TLR9−/− and MyD88−/− (Department of Host Defense, Research Institute for Microbial Diseases, Osaka University and Core Research for Evolutional Science and Technology, Japan Science and Technology Corporation, Suita) and MARCO−/− (provided by Dr Andrij Holian at the University of Montana) strains as previously reported^62^. Human monocytes were isolated from the peripheral blood of normal human volunteers (blood donors at local blood bank) and were differentiated into mature macrophages as previously described^63^. Cells were cultured at specific densities as described in the text. The mouse macrophage cell lines IC21 and RAW 264.7 were purchased from the American Type Culture Collection (Rockville, MD) and were cultured in RPMI 1640 medium (Gibco BRL, Rockville, MD) supplemented with 10% fetal bovine serum, 100 U ml^−1^ of penicillin G and 100 μg ml^−1^ streptomycin and grown at 37 °C in 5% CO_2_.

### Experimental silicosis

Crystalline silica (α-quartz, average size, 1.7 μm) was obtained from US Silica Co. (Berkeley Springs, WV) and was selected by sedimentation according to Stokes’ law, acid hydrolysed and baked overnight (200 °C, 16 h) to inactivate endotoxin contamination. Ten- to twelve-week-old Specific pathogen-free female C57BL/6 mice (Charles River Laboratories, Kingston, NY) weighing 20–25 g were housed in pathogen-free cabinets. Animals were anaesthetized via intraperitoneal administration of sodium pentobarbital (200 mg kg^−1^; Henry Schein, Indianapolis, IN), exposed to silica (0.2 g kg^−1^) or saline (control) and after 3 days received intravenous injections of human MSC or fibroblasts (500,000 cells per mouse), or human MSC-derived exosomes (∼3 × 10^11^ exosomes containing 40 μg protein). Animals were euthanized by exsanguinations at 3, 14 and 28 days post exposure. All lung samples were harvested at a terminal anaesthesia point, fixed and embedded in paraffin for histological analysis or snap-frozen in liquid nitrogen and stored at −80 °C for RNA or collagen analysis. All procedures involving animals were approval by the Institutional Animal Care and Use Committee of the University of Pittsburgh.

Collagen deposition was measured with hydroxyproline assay as previously described^64^. Briefly, dried left lungs were acid-hydrolysed in 6 N HCl at 110 °C under nitrogen gas. After evaporation of HCl, samples were suspended in PBS and incubated in a 60 °C water bath. Following three consecutive high-speed centrifugations, a 40 × dilution in PBS of the supernatant was oxidized with chloramine-T and the reaction was stopped with perchloric acid. Finally, *p*-dimethylaminobenzaldehyde was added and samples were analysed spectrophotometrically at 557 nm. Hydroxyproline content per lung was calculated from a hydroxyproline standard curve.

Inflammatory cells were measured in BALF of mice 28 days after the treatment with silica or intervention with MSCs, MSC-derived exosomes or fibroblasts. Mice were killed with an overdose of sodium pentobarbital and the lungs lavaged with a single instillation of 1 ml normal saline. Recovery of BALF was consistently above 80%. BALF cells were counted using a Z1 Coulter Particle Counter (Beckman Coulter Inc., Fullerton, CA), and 50,000 cells were transferred to glass slides using a Shandon Cytospin 4 (Thermo Electron Corporation, Pittsburgh, PA) at 750 r.p.m. for 5 min. After 3 days of drying, cells were stained using the Diff-Quick stain (Dade Behring, DE), and macrophages, neutrophils and lymphocytes were counted (400 cells).

### Isolation of MVs

Purification of exosomes was performed as previously described^17^,^18^. Briefly, MSCs were expanded in multilayer tissue culture plates (Millipore) in medium depleted of serum derived-MVs/exosomes by overnight ultracentrifugation at 100,000 *g* or in serum-reduced MSC medium (Invitrogen) supplemented with bovine serum albumin (Sigma-Aldrich). Conditioned medium from 10 × 10^6^ MSCs was collected every 24 h and was subjected to successive centrifugations at 300 and 2,000 *g* (10 min each) to remove cells. The cell-free supernatant was then centrifuged at 10,000 *g* for 30 min to remove cell debris and 100,000*g* (Beckman Coulter Optima L-90K ultracentrifuge) at 4 °C for 90 min. The 10,000 and 100,000 *g* pellets were washed and suspended in 100 μl of PBS. Total protein was isolated from 2–5 × 10^6^ MSCs and quantified using the Bradford protein assay (Thermo Fisher Scientific). The protein content of exosomes (100,000*g*) or vesicles (10,000 *g*) recovered from conditioned medium was quantified (∼3–5 μg per preparation) and pooled to perform electron microscopy, western blotting or *in vivo* administration.

Vesicles isolated from conditioned medium from 10 × 10^6^ human MSCs were pooled (five to seven preparations) and overlaid on sucrose as previously described^16^,^17^,^18^. Exosomes were suspended in 2 ml of 2.5 M sucrose and loaded in a SW41 tube, and 10 ml of a continuous sucrose gradient, from 2 to 0.25 M, was layered on top. Tubes were centrifuged for 18 h at 4 °C at 100,000 *g* (ref. 18). Single 1-ml fractions were collected and sucrose density was determined on an aliquot of each gradient with the use of a refractometer (Reichert) and the fractions re-suspended in 3 ml PBS, centrifuged for 70 min at 100,000 *g* 4 °C and the pellet suspended in sample buffer before being analysed with NanoSight tracker to determine particle number as previously described^19^, or SDS–PAGE and western blot.

### Measuring MSC-derived exosomes by nanoparticle tracking

Exosomes were diluted in particle-free PBS and measured using Nanoparticle Tracking Analysis as described^19^. Briefly, videos were collected using a NanoSight LM10 system equipped with a 405-nm laser and a highly sensitive digital camera (OrcaFlas2.8, with camera shutter speed fixed at 30.01 ms and gain set to 500), and analysed with the NTA software (NANOSight version 2.3). Ambient temperature was recorded manually and samples administered and recorded under control flow, using NanoSight syringe pump and script control system. For each sample, three videos of 60-s duration were obtained with 10-s delay between recordings, generating three replicate histograms. The typical number of tracks per sample was ∼1,500. To obtain average particle counts, the area under the curve was calculated using the Prism-4 software. Black histograms indicate the average size concentration, and red error bars indicate the±1 s.e. of the mean.

### Western blotting and histochemistry

Western blot analysis was conducted as previously described^44^. The following primary (at a concentration of 1.5 μg ml^−1^) and secondary (0.5 ng ml^−1^) antibodies were used for western blotting: polyclonal anti-ARRDC1, anti-LC3, anti-MDA5, anti-TLR9 (Abcam), polyclonal anti-Atg-12 (Santa Cruz Biotechnology, SC-68884), polyclonal anti-Pink1 (Santa Cruz Biotechnology, SC-33796), anti-Parkin mouse monoclonal (clone PRK8, Santa Cruz Biotechnology), polyclonal anti-Miro (Santa Cruz Biotechnology, SC-292547), anti-CD9 (MM2/57, Millipore), anti-CD63 (MEM-259, ThermoFisher), anti-TSG101 (51/TSG101, BD Biosciences), anti-Human MFGE8 (Abnova), anti-Dicer (D11, Santa Cruz Biotechnology, used at a concentration of 2 μg ml^−1^), anti-Tubulin, anti-LC3 (rabbit polyclonal), anti-TLR-7 (Cell Signaling Technology), Beta actin (AC-15, Sigma-Aldrich) and anti-GAPDH (Santa Cruz Biotechnology, SC-25778). NF-κB nuclear translocation was characterized by staining cells with anti NF-κB-P50 Alexa Fluor 488 (E-10, Santa Cruz Biotechnology). Images were obtained using a Leica TCS NT upright confocal microscope.

### Live cell imaging and electron microscopy

Human or mouse MSCs (1 × 10^6^) were infected with baculoviruses (BacMam2 delivery system, Life Technologies) encoding fluorescent proteins (Organelle Lights, Life Technologies) to target mitochondria (GFP) or LC3-associated with phagosomes (RFP) for 48 h at 37 °C at an multiplicity of infection of 30 in accordance with the manufacturer’s specifications. Subsequently, 1 × 10^5^ cells were plated into Mattek dishes and co-incubated with an equal amount of bone marrow-derived macrophages (C57BL/6J, BALB/CJ and FVB/NJ) or macrophage cell lines (RAW 264.7 or IC-21). MSCs were maintained alive using a Tokai Hit temperature-controlled humidified chamber at 37 °C for periods of up to 72 h. During this time, multimode imaging events in five dimensions were obtained using high-speed confocal imaging on a Nikon Ti stand equipped with a Prairie Sweptfield scanner and Elements software. Images were collected using shuttered illumination in two colours with cubes specifically designed for GFP, ratiometric dyes or membrane/cytoplasmic labels, and differential interference contrast modes. Image series were viewed as movies for qualitative assessment, or were imported to Metamorph for quantitative assessment of cell motion, protein expression and organelle transfer between MSCs and macrophages. To inhibit the mitophagic flux, double GFP and RFP-labeled MSCs were treated with 3 μM chloroquine (Invitrogen) and images taken every 5 min as described. In some experiments, MSCs were stained with Cy5 dye (Amersham) following the manufacturer’s instructions; MSC-conditioned medium was collected and extracellular MVs isolated as described above. Cy5-labelled exosomes/MVs were co-cultured on Mattek dishes with macrophages from different mouse strains (C57BL/6J, BALB/CJ, TLR4−/−, TLR9−/−, MyD88−/−, MARCO−/− and RAW 264.7) and phagocytosis followed as described above. To inhibit phagocytosis, macrophages were treated for 2 h with 100 μg ml^−1^ dextran sulfate (Sigma-Aldrich) before their exposure to Cy5-labelled vesicles.

Electron microscopy analysis was performed on MSC extracellular vesicles loaded on carbon-coated grids and fixed in 4% paraformaldehyde. Grids were labelled with mouse or rabbit anti-human ATP synthase (ab54880). Grids were observed at 80 kV with a JEOL 1210 transmission electron microscope (TEM) with high-resolution AMT digital camera. Size of MVs was measured on five to seven different pictures using the TEM software.

### RNA analysis

RNA was extracted using the RNeasy micro kit (Qiagen), and first-strand cDNA synthesized using SuperScript III (Invitrogen). Quantitative PCR was performed using the Taqman single-gene expression assay (Invitrogen) on an ABI 7900 Real Time PCR System (Applied Biosystems). The following oligonucleotide primers were used: hCOX-I (Hs03929097_g1), hGAPDH (Hs02596864_g1), mCOLLAGEN (Mm00472589_m1), mTNF (Mn 00443258_m1), mIL-6 (Mm00446190_m1), mIL-10 (Mm00439614_m1), mMyD88 (Mm00440338_m1), mTLR9 (Mm00446193), mTLR7 (Mm00446590_m1) and mTLR4 (Mm00445273_m1). Mouse Toll Like Receptor Signaling Pathway genes were measured using a mouse RT^2^ Profiler PCR Array System (SABioscience Version 4) as previously described. Alternatively, total RNA was isolated from five different human MSC donor populations or their exosomes using the Qiagen miRNeasy Mini Kit (PN 217004) according to the manufacturer’s instructions. Samples were then analysed using human microRNA microarrays from Agilent (Version 3, PN: G4470C) and data analysed using the Agilent GeneSpring GX 11 software. In some cases, array data were validated via quantitative RT–PCR.

mtDNA from five human MSC donors was isolated, and human COXI, tRNA leucine and mitochondrial hypervariable regions I and II (HVRI) were sequenced to generate specific probes to allow for the documentation of fragment length polymorphism^30^. We identified polymorphisms in the sequences of HVR in each one of the five hMSC donors used in the current study and partial homology in human tRNA^leu^ probes between species that yielded a positive hybridization signal in mouse macrophages. Subsequently, we sequenced the *COX* gene from our five human MSC donors and designed COX I-specific primers encompassing base pairs 64–293 that were conserved among the different human MSC populations. In Supplementary Fig. 4 we illustrate sequences spanning nucleotides 108–293 that exhibit little homology with the mouse gene and contain a BfaI restriction site (5′-CTAG-3′) allowing the generation of a specific restriction map. PCR products were digested with BfaI and products resolved with agarose electrophoresis (Novex, Invitrogen).

### Fluorescent activated cell sorting

Mouse lung tissue was digested with collagenase-dispase (Sigma-Aldrich) to generate a single-cell suspension as previously described^50^. Cells were then stained with the following antibodies: Anti-F4/80-FITC (BM8, Abcam), CCR2-PE (475301, R&D System), Ly-6C-APC (HK1.4, eBiosciences) and CD11b-V450 (M1/70, BD Biosciences). CD11b^+^ and Ly6C^hi^ cells were gated within the F4/80^+^ subpopulation, and from these CCR2^+^ cells were sorted as previously described^50^. Characterization of exosomes markers was performed as described previously^8^,^17^. Aldehyde/sulfate latex beads (Life Technology) were coupled with 30 μg of exosomes, stained with fluorescein isothiocyanate -conjugated anti-CD63 (H5C6, BD Pharmingen) and analysed using FACS. Macrophages co-incubated with Orange Fluorescent Protein (OFP)-mitochondria-labelled MSCs were incubated with anti-F4/80^+^ and double-positive F4/80^+^/RFP cells sorted using FACS. Analysis was performed using a FACS Canto II (BD Biosciences) and data analysed using the FlowJo v7.6.3 software.

### Mitochondrial bioenergetics

Mitochondrial respiration in macrophages (3–5 × 10^8^) was determined by measuring the OCR using an XF24 Extracellular Flux Analyzer (Seahorse Bioscience). Macrophages were seeded at a density of 25,500 cells per well on the customized Seahorse 24-well plates. Cells were incubated in XF24 assay media for 1 h. OCR was calculated by plotting the O_2_ tension of the media in the chamber as a function of time (pmol min^−1^). Values were normalized to total protein content of each well. Data were collected from an average of six independent wells and experiments were conducted in triplicates. Where indicated, MSCs were stained with MitoSOX-Red (Invitrogen) to quantify mitochondrial superoxide production as previously described^28^. For microscopic analysis, 10 μM MitoSOX-Red was used, while 100 nM was used for fluorescent quantification. To confirm mitochondrial localization of the dye, cells were counterstained with 5 μM Mito-Tracker Green (Invitrogen) for 10 min. Normalization of fluorescent data was performed using counterstaining with Hoechst 33342 (1 μM) for 5 min. Fluorescent wavelength pairs for the individual dyes were 510 nm/580 nm for MitoSOX-Red, and 490 nm/516 nm for Mito-Tracker Green. To determine mitochondrial membrane potential, cells were grown to ∼80% confluence in 96-well plates and stained with 2 μM JC-1 for 10 min following anisomycin treatment. Green fluorescence (depolarization) was monitored at 488 nm and red fluorescence (polarized) at 590 nm on a Spectromax M5e plate reader (Molecular Devices Inc., Sunnyvale, CA, http://www.moleculardevices.com). Actively growing cells were used as a negative control, and cells treated with 2 mM carbonyl cyanide *p*-trifluoromethoxyphenylhydrazone (FCCP) and 1 mM valinomycin were used as the positive control. Normalization to cell number was achieved as described above. Data are presented as per cent viable cells based on presumed 95–100% depolarization observed in FCCP/valinomycin-treated cells^37^.

### DICER knockdown in human MSCs

Human MSCs (1 × 10^6^) were infected with a lentivirus encoding a DICER-specific shRNA l (Santa Cruz Biotechnology) in complete αMEM containing 5 μg ml^−1^ Polybrene overnight at 37 °C 5% CO_2_. Clones expressing the virus were selected by their resistance to Puromycin (Sigma-Aldrich) and reduced DICER expression in selected cells was confirmed by western blotting using an anti-hDICER antibody (H-212, Santa Cruz Biotechnology).

### Statistics

Results are presented as mean±s.e.m. from at least three experiments. Statistical differences between groups were analysed using one-way ANOVA followed by SNK test for *post hoc* pairwise comparisons (statview 4, Abacus Concenpt Inc, Berkely, CA). For microarray data, the significant differences were determined by one-way ANOVA with Holm–Sidak all pairwise multiple comparison procedure. The statistical significance of differences was set at *P*<0.05.

## Additional information

**Accession codes:** Microarray data have been deposited in the GEO NCBI database with accession code GSE71241.

**How to cite this article:** Phinney, D. G. *et al*. Mesenchymal stem cells use extracellular vesicles to outsource mitophagy and shuttle microRNAs. *Nat. Commun.* 6:8472 doi: 10.1038/ncomms9472 (2015).

## Supplementary Material

Supplementary Figures Supplementary Figures 1-4

Supplementary Movie 1 Human MSC load mitochondria into LC3-labelled vesicles. Live cell imaging demonstrating that under normal culture conditions human MSC mitochondria are loaded in the cytoplasm into LC3-labelled vesicles and subsequently migrate towards the periphery of the cell where they are incorporated into outward budding blebs in the plasma membrane.

Supplementary Movie 2 Inhibition of mitophagy flux induces MSC apoptosis. Apoptosis of human MSCs cultured under normal serum conditions in the presence of low (3-5 μM) concentrations of chloroquine, which prevents lysosomal degradation and blocks the mitophagy flux of mitochondria in mitophagosomes.

Supplementary Movie 3 Human MSCs outsource mitophagy to macrophages. Live microscopy reveals that macrophages nibble the plasma membrane of human MSCs establishing cell contact at areas where membrane blebs are enriched in LC3 labeled vesicles, which are subsequently stripped by the macrophages.

Supplementary Movie 4 MSCs transfer mitochondria to macrophages. Cultured human MSCs establish physical interaction to transfer their GFP-labeled mitochondria, observed in filamentous form, to mouse macrophages.

Supplementary Movie 5 MSCs rapidly transfer mitochondria to macrophages. Cultured human MSCs establish physical interaction to rapidly transfer their GFP-labeled mitochondria to mouse macrophages. GFP signal is overcompensated to allow the tracking of the GFP-labeled mitochondria inside the acidic pH of the macrophage.

Supplementary Movie 6 Macrophages avidly phagocytose MSC-derived extracellular vesicles. Live cell imaging illustrating phagocytosis of extracellular vesicles by macrophages over a period of 18 minutes. Confocal images confirm that the engulfed Cy5-labeled vesicles resided within the cell body of the macrophages.

Supplementary Movie 7 Dextran Sulfate inhibits phagocytosis of hMSC-derived extracellular vesicles. Pre-incubation of macrophages with non-specific inhibitors of phagocytosis, such as dextran sulfate (100 μg/ml), inhibited the entrance of Cy5-labeled hMSC-derived extracellular vesicles inside the macrophage, which accumulated on the macrophage surface where they formed a cap.
