# Supplementary material for: Mesenchymal stem cells use extracellular vesicles to outsource mitophagy and shuttle microRNAs
Source: Nat Commun. 2015 Oct 7;6:8472. doi: 10.1038/ncomms9472 (PMC4598952; doi:10.1038/ncomms9472)
Supplement: Supplementary Figures — 1-4 [file ncomms9472-s1.pdf]

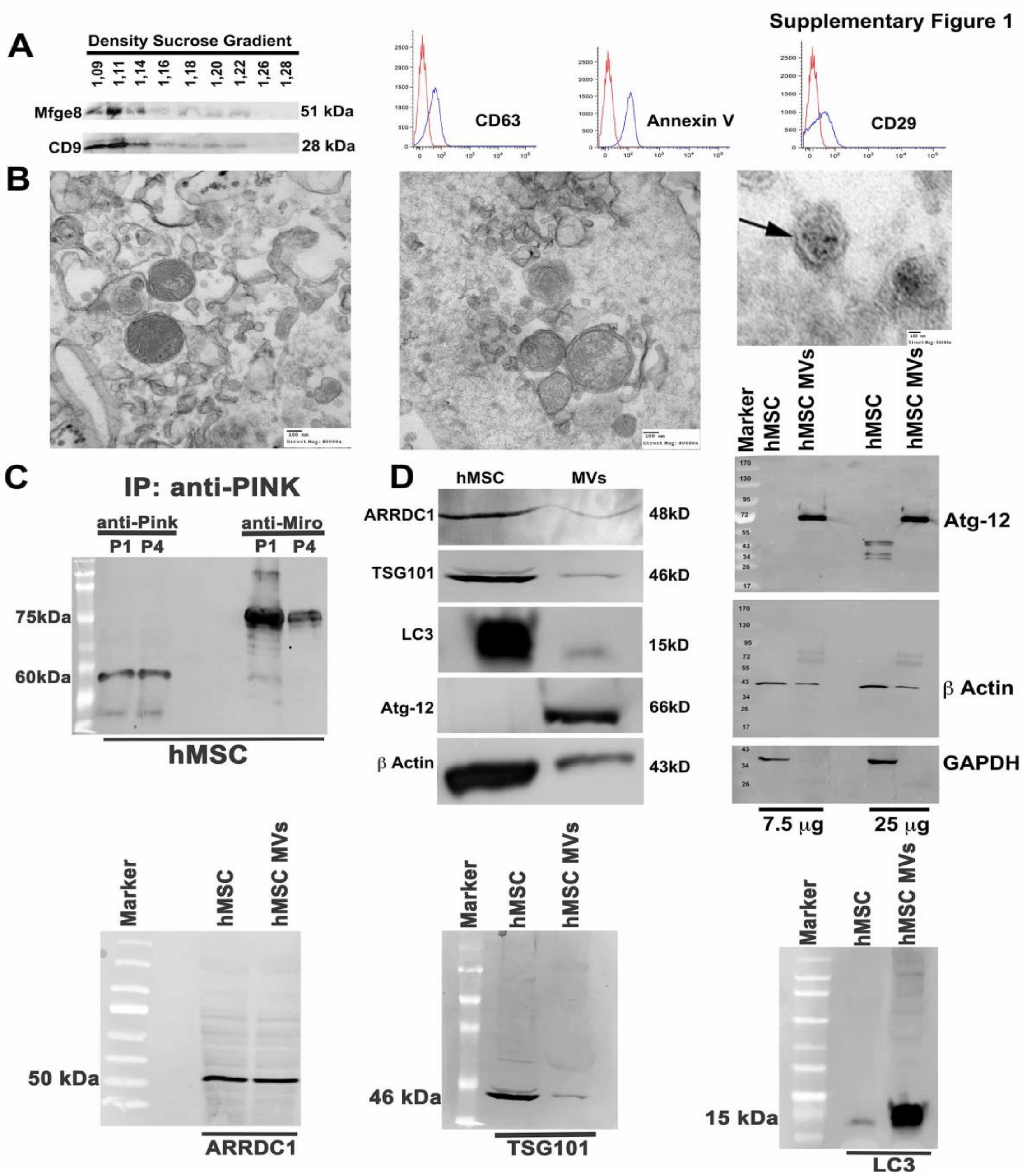

Supplementary Figure 1. Characterization of MSC-derived extracellular vesicles: A) Left panel**,** Western blot of human MSC-derived exosomes isolated from conditioned medium by differential ultracentrifugation (100,000g/18h) through a 30% sucrose gradient. Expression of CD9 and Mfge8 was highest in sucrose fractions of 1.11 g/ml and 1.14g/ml density as determined by refractometry. Right panel, FACS analysis of exosomes shows expression of CD63, Annexin V, and CD29. **B)** Electron microscopy of human MSC-derived MVs recovered from conditioned medium by low speed centrifugation (10,000 g/1h) show multiple vesicles >100 nm in size (left and middle panels) that express the mitochondrial specific proteins ATP synthase based on immuno-gold staining (arrow, right panel). **C)** Western blot showing immune-precipitation of Miro using anti-Pink1antibody (Santa Cruz Biotechnology, SC-33796 used at 2 μg for every 100 μg of protein) from mitochondrial extracts prepared from human

MSCs expanded in 21% oxygen at P1 or P4. The magnitude of this interaction is diminished as a function of passage due to activation of Pink kinase and reduced expression of Miro in the mitochondria. **D)** Western blot of proteins isolated from human MSCs or human MSC-derived MVs showing expression of Atg-12, ARRDC1, TSG101, and LC3. Based on equal loading of proteins it is clear that MVs express much lower levels of LC3 as opposed to whole cells whereas Atg-12 is highly enriched in human MSC-derived MVs. MVs also express lower levels of beta-actin and GAPDH as compared to human MSCs.

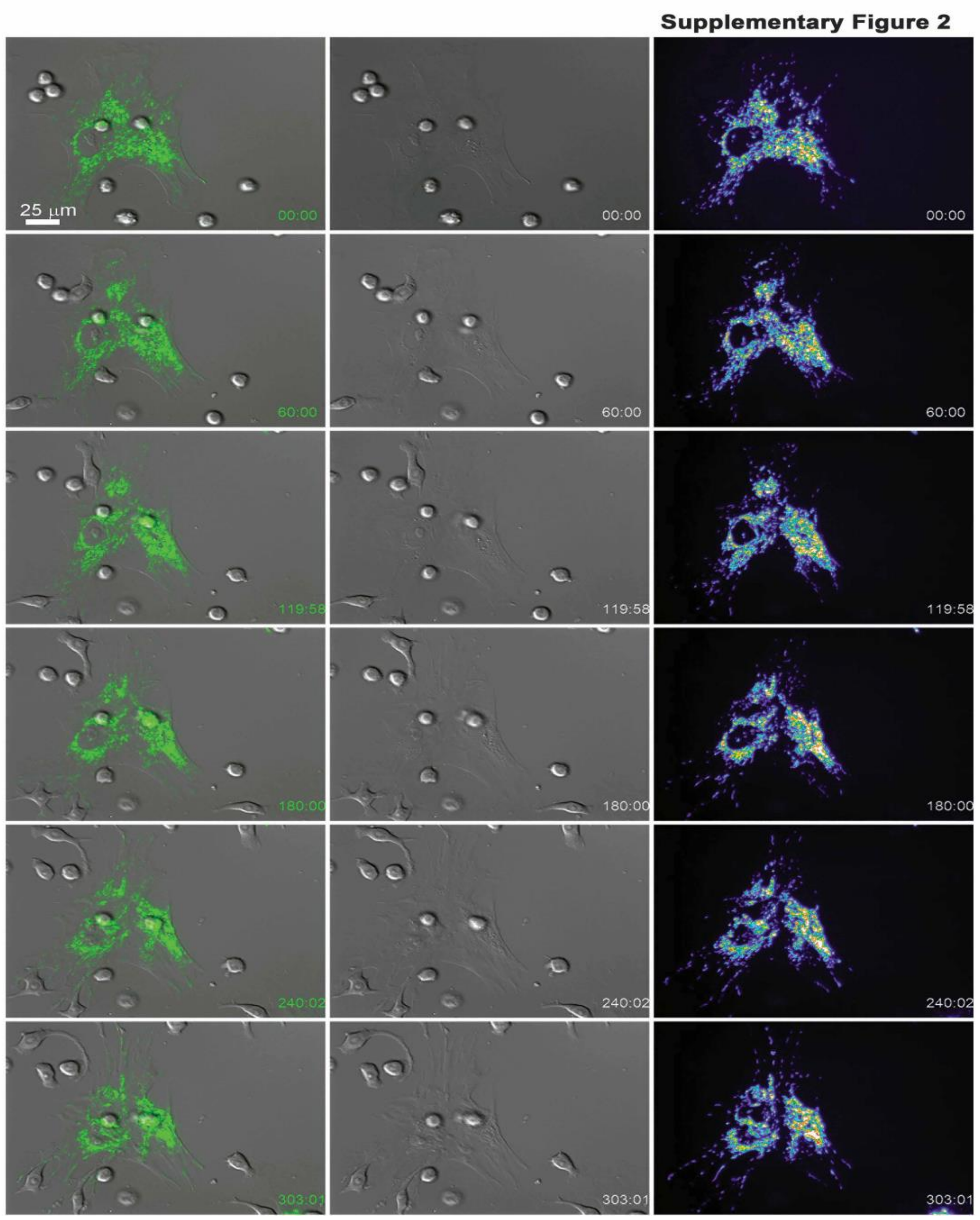

Supplementary Figure 2. Fibroblasts do not transfer mitochondria to macrophages. Time-lapse images (60 minute intervals) of human dermal fibroblasts co-cultured with

macrophages over a 5h period reveals no evidence of mitochondrial transfer. The left panel shows overlay of differential interference contrast (DIC, middle panel) and fluorescence images (far right). Fibroblast mitochondria appear green. In the panel on the far right, the mitochondrial fluorescence has been pseudo-colored to reveal subtle differences in intensity that may not be readily apparent in the overlay (far left). There was no evidence of mitochondrial transfer (i.e. no appearance of mitochondrial fluorescence) from the fibroblasts to the macrophages. This data is representative of 3 separate experiments. Scale bar = 25 microns.

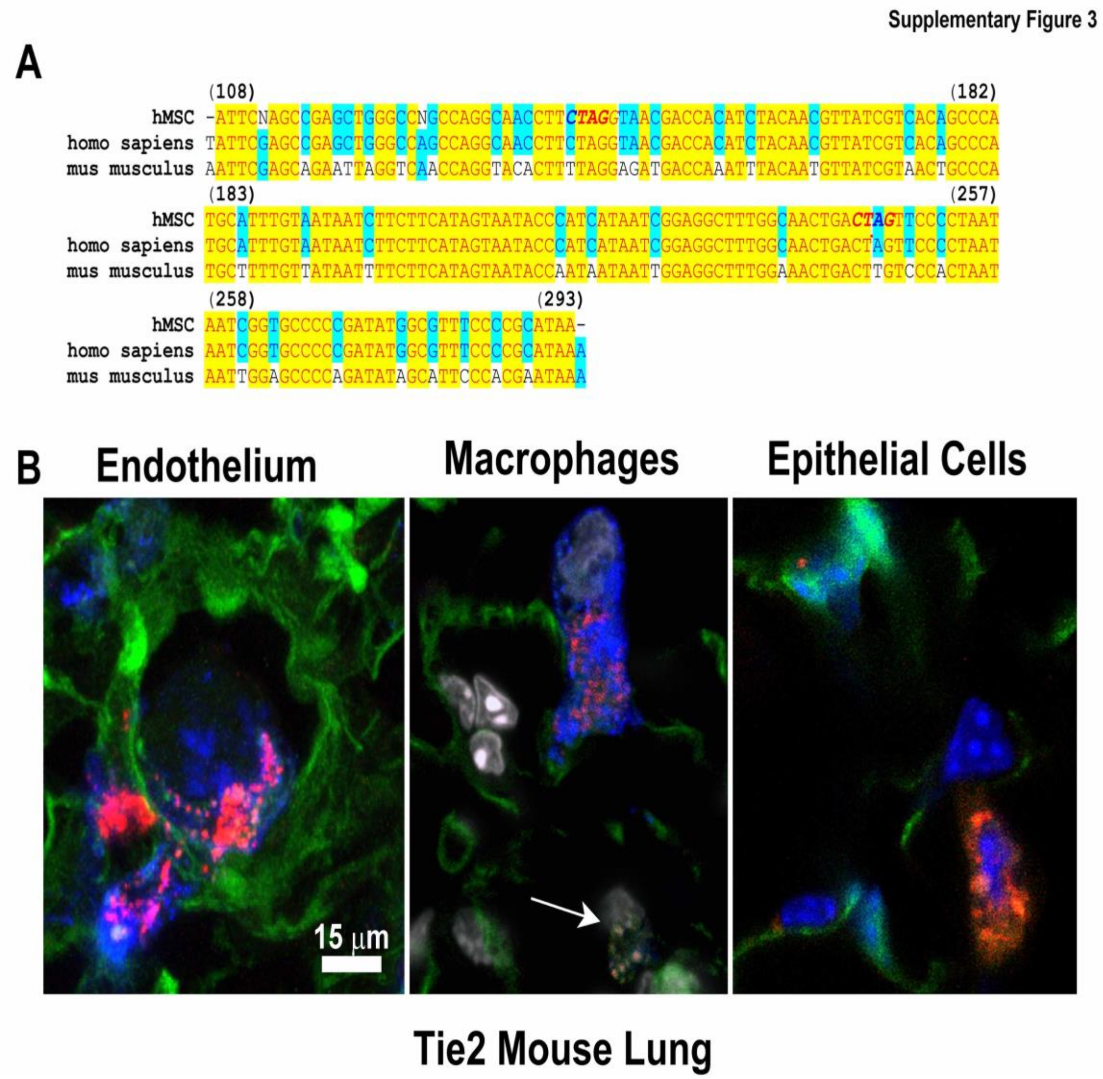

Supplementary Figure 3. MSC transfer mitochondria in vivo to the mouse lung. A) Partial sequence of the COX I gene obtained from a human MSC donor is highly homologous to the published sequence for the human gene but shares minimal homology with the mouse gene. Non-homologous regions are illustrated by blue, and white boxes, and the BfaI restriction sites are bolded. **B)** High power image (100x) of the endothelial and alveolar region of lung tissue from a Tie2 mouse harvested 24h after administration of of human MSCs infected with Organelle Lights to label mitochondria (red). The presence of RFP-labeled mitochondria inside epithelial, macrophages (white arrow), and endothelial cells is clearly evident. Scale bar = 15 microns.

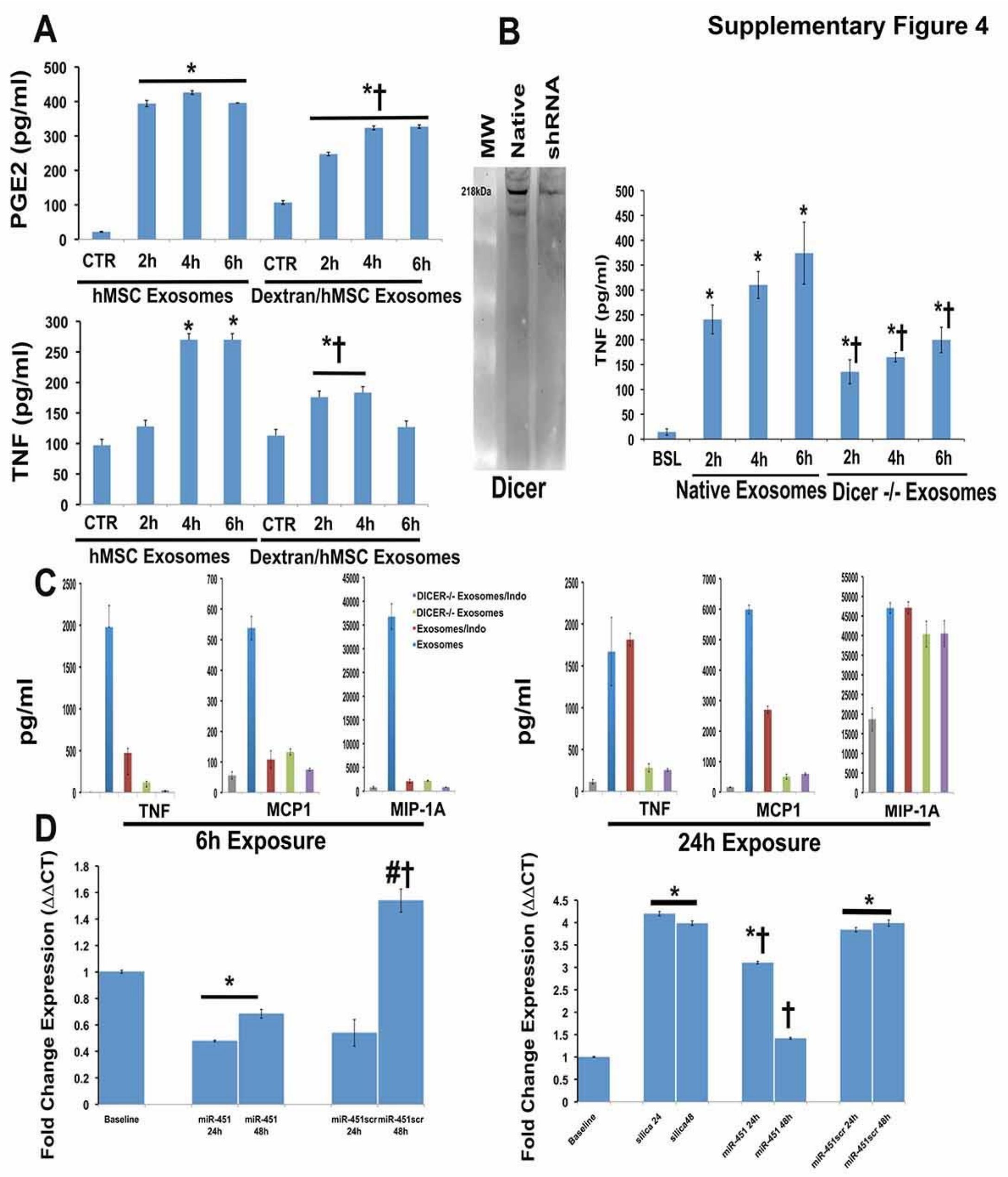

Supplementary Figure 4. TLR signaling in macrophages is suppressed by microRNAs packaged within human MSC-derived exosomes. A) RAW 264.7 macrophages pretreated with dextran sulfate exhibit significantly reduced PGE2 (upper panel) and TNF (lower panel) secretion following treatment with exosomes. (*$p<0.05$ compared to control treated, † *$p<0.05$ compared to exosome treated cells without dextran incubation, Students t test). **B)** Left panel,

Western blot, using D11 antibody, Santa Cruz Biotechnology, at a concentration of 2 μg/ml, showing knockdown of DICER in human MSCs following transfection with a DICER-specific shRNA. Right panel, ELISA of TNF secretion by macrophages as a function of time post-treatment with human MSC-derived native exosomes or exosomes from human MSCs transduced with a Dicer shRNA (*p<0.05 compared to baseline,#p<0.05 compared to the effect of native exosomes by Student t-test). **C**) Luminex analysis of TNF, MCP1 and MIP-1A secretion by RAW 264.7 macrophages at 6h (left panel) or 24h (right panel) post-treatment with human MSC-derived exosomes from (B) in the presence or absence of indomethacin. **D)** ELISA of TNF secretion by RAW 264.7 macrophages transfected with a miR-451 mimic or scrambled siRNA (left panel) or after exposure to silica particles for 24 or 48 h (right panel). Plotted values (mean ± SEM) are from experiments repeated three times. (*p<0.05 compared to control treated, † *p<0.05 compared to scrambled siRNA treated cells, Students t test).
